# Preliminary Study on Hourly Dynamics of a Ground-Dwelling Invertebrate Community in a Farmland Vineyard

**DOI:** 10.3390/insects15010027

**Published:** 2024-01-02

**Authors:** Meixiang Gao, Jiahuan Sun, Tingyu Lu, Ye Zheng, Jinwen Liu

**Affiliations:** 1 Department of Geography and Spatial Information Techniques, Ningbo University, Ningbo 315211, China; gaomeixiang@nbu.edu.cn (M.G.); 2111073020@nbu.edu.cn (J.S.); 2 Zhejiang Collaborative Innovation Center & Ningbo Universities Collaborative Innovation Center for Land and Marine Spatial Utilization and Governance, Ningbo 315211, China; 3 College of Geography and Environmental Sciences, Hainan Normal University, Haikou 571158, China; hrblutingyu@163.com; 4 Faculty of Electrical Engineering and Computer Science, Ningbo University, Ningbo 315211, China; zhengye@nbu.edu.cn; 5 Institute of Plant Protection, Jilin Academy of Agricultural Sciences, Changchun 130033, China

**Keywords:** subtropical region, infrared camera trap, hourly variation, millipede, slug, beetle, grasshopper, ant

## Abstract

**Simple Summary:**

Understanding diel variations in ground-dwelling invertebrates is an important issue for agricultural ecology, which has been extensively studied at the population level. However, contemporary studies on hourly variations in the field are scarce at the community level owing to the lack of an effective method for collecting hourly data. Therefore, this study used a novel, automatic method using infrared camera traps to photograph a ground-dwelling invertebrate community at 5 min intervals from 31 July to 29 September in farmland in Ningbo City, China. In total, 9 taxa and 1147 individuals were identified across 42 d in 217,728 photographs. The taxonomic richness and abundance of the ground-dwelling invertebrate community did not show significant hourly variations within each day nor significantly preferred active hours. Only millipedes had significant hourly variations within each day, with a significant preference for activity between 1:00 and 4:00. Additionally, slugs, beetles, and grasshoppers showed preferred active hours around sunset and midnight. This study provides a useful method for monitoring hourly changes in ground-dwelling invertebrate communities in farmlands as well as fine temporal resolution data to understand the responses of ground-dwelling invertebrate communities to climate change and human management.

**Abstract:**

We evaluated the hourly dynamics of ground-dwelling invertebrate communities in farmland using infrared camera traps between August and September 2022. No significant variations within 24 h nor between any two time points of each day were observed in the taxonomic richness and abundance of the entire community. However, the periods from 4:00 to 7:00 and 13:00 to 20:00 showed relatively high taxonomic richness, while those from 2:00 to 6:00 and 16:00 to 21:00 showed relatively high abundance. Millipede abundance varied significantly in a 24 h period, with higher abundance from 3:00 to 4:00 and 1:00 to 2:00. Additionally, slug, beetle, and grasshopper abundances were significantly higher from 22:00 to 23:00, 17:00 to 18:00, and 23:00 to 24:00, respectively. The abundance of other taxa did not show significant variations between any two time points of a day. Predominant generalist predators showed positive correlation in their activity times. These results suggest that significant variations within each 24 h period are uncommon at either community or taxa (except for millipedes) levels in farmland ground-dwelling invertebrates. Further, while most taxa had significantly preferred active hours, the total community did not. Therefore, hourly dynamics should be considered to understand biodiversity maintenance.

## 1. Introduction

The diel dynamics of invertebrates (diel activity), that is, their behavior throughout the 24 h period of a day, vary among and within populations. Some invertebrate populations are active across a variety of periods, whereas others are active at one peak or a few brief peaks [1,2]. Invertebrate diel dynamics reflect their adaptations, requirements, and interactions [3]. Several studies have focused on the diel dynamics of invertebrate species/populations in laboratories under controlled conditions [4] and field investigations [5]. These have included research on ants (*Crematogaster matsumurai*) [6], cockroaches (*Blattella germanica*) [7], ticks (*Ixodes ricinus*) [8], and crickets (*Gryllus campestris*) [9]. However, compared to the species/population level, the diel dynamics of invertebrates at the community level remain poorly studied, especially those of ground-dwelling invertebrate communities.

Ground-dwelling invertebrate communities play important roles in the biogeochemical processes in soil ecosystems [10,11]. Identifying the diel dynamics of ground-dwelling invertebrate communities is critical for understanding their ecology and function [12], responses to climate change and human management [13], roles in pest-controlling strategies [14], and maintenance mechanisms [15] in ecosystems. The results of a study on an agricultural ecosystem have shown that ants, springtails, micro Diptera, and tumbling flower beetles are diurnal taxa, whereas Elateridae and Aphididae tend to be nocturnal taxa [16]. The activity and abundance diel patterns of ground-dwelling collembolan communities have been reported to be highest from 12:00 to 00:00 and lowest from 00:00 to 06:00 in wheat farmland [17]. Furthermore, diel patterns in the activity of carrion-visiting Coleoptera communities were observed, with more individuals active during the day than at night in a grass meadow [18]. Although these studies focused on the diel dynamics of ground-dwelling invertebrate communities, the hourly variations within 24 h of a day are largely unknown owing to a lack of dynamics data at an hourly temporal resolution in fields.

Monitoring ground-dwelling invertebrate communities in farmland fields at an hourly temporal resolution is challenging. Previous studies have usually used suction samples, sweep net samples [17], light sources [19,20], and pitfall traps [18,21] to collect diel dynamics data of ground-dwelling invertebrate communities. However, these methods are time-consuming, labor-intensive, and costly [22], usually requiring relatively coarse temporal intervals, such as 3 [23], 4 [24], 6 [17], or 12 h [25] intervals. Therefore, it is still unclear whether significant variation exists within a 24 h period in ground-dwelling invertebrate communities at 1 h intervals. Infrared camera traps (ICTs), which are automated, powerful, environmentally friendly methods with fine temporal resolution, are considered to be useful for collecting invertebrates from forest [26], grassland [27], and farmland ecosystems. Using ICTs can enable the collection of large amounts of invertebrate data at fine temporal resolutions (min, h, d) [27,28]. Therefore, this study used ICTs to photograph a ground-dwelling invertebrate community with an hourly temporal resolution.

The objective of this study was to understand the hourly variation in the taxonomic richness and abundance of a ground-dwelling invertebrate community over a daily 24 h period. We monitored a ground-dwelling invertebrate community in a farmland field in Ningbo City, southeastern China, over 42 d (1008 h). The results of this study will provide a useful method for monitoring ground-dwelling invertebrate communities at an hourly temporal resolution and contribute to understanding their responses to climate change and human management at fine temporal resolutions.

## 2. Materials and Methods

### 2.1. Study Site

This study was conducted in Ningbo City (28°51′–30°33′ N, 120°55′–122°16′ E), Zhejiang Province, Eastern China. The topography in Ningbo undulates from lowlands to mountainous areas, with plains accounting for 40.3% of the area. The urban and suburban areas are located 4.0–5.8 m and 3.6–4.0 m above sea level, respectively. Ningbo has a subtropical monsoon climate, with an average annual temperature of 16.4 °C and precipitation of 1480 mm [29].

### 2.2. Setting ICTs, Collecting Data, and Identifying Invertebrates

Data were collected in a vineyard at Tiansheng Farm (29°80′ N, 121°40′ E). Six plots were established in the vineyard, each spanning 234 m^2^. An ICT (BG636-48M; Boly Media Communications, Santa Clara, CA, USA) was placed at the center of each plot, each mounted on a fixed support consisting of three stainless steel tubes and powered using eight lithium batteries and a solar panel. The camera lens was positioned parallel to and 40 cm above the soil surface. Each infrared camera captured three consecutive images at 5 min intervals. Invertebrates that entered and were active in 20 × 20 cm^2^ squares set directly under each ICT were photographed. The ICTs worked continuously in the field from 20:00 on 31 July to 24:00 on 29 September 2022. Except for the period affected by typhoons, the ICTs worked for a period of 42 d (1008 h), photographing 217,728 images. All photographed invertebrates were classified into different taxa according to a flowchart for identifying ground-dwelling invertebrates using ICTs and information from a previous publication [30].

### 2.3. Data Analysis

Taxonomic richness (number of taxa) and abundance (number of individuals) were determined to evaluate the diversity of the total ground-dwelling invertebrate community captured by the ICTs. The taxonomic richness and abundance at any certain hour of the day were the sum of those in the previous 60 min. For example, the abundance at 10:00 was the sum of abundances detected between 9:00 and 10:00. One-way analysis of variance and post hoc least significant differences were used to test the differences in taxonomic richness and abundance of the total ground-dwelling invertebrate community and that of each taxon within a daily 24 h period, followed by multiple-testing correction for the false discovery rate [31]. Statistical significance was defined at *p* < 0.05. A Pearson correlation coefficient was employed to assess the relationships between various taxonomic abundances in 24 h at 1 h intervals. These relationships were computed using the ‘cor’ function, and a correlation plot was generated through the ‘corrplot’ function in the ‘corrplot’ package in R software 4.3.1, with a significance level of *p* < 0.05 [32]. In Pearson analysis, the corresponding values are Pearson correlation coefficients *r*, which range from −1 to 1. Additionally, a value of *r* less than 0 indicates a negative correlation, while a value of *r* greater than 0 indicates a positive correlation. A linear mixed-effect model was used to evaluate the effects of monitoring Hour (i.e., 1:00 to 24:00) and monitoring Day (i.e., the 1st day to the 42nd day) on taxonomic richness and abundance of the total ground-dwelling invertebrate community and abundance of each taxon using the ‘lmer’ function in the ‘lme4’ package [33]. The fixed effects included monitoring Hour and monitoring Day, while the random effect was the camera trap (i.e., plot). All statistical analyses were performed using R software 4.3.1 [34].

## 3. Results

### 3.1. Diel Dynamics in the Richness and Abundance of the Ground-Dwelling Invertebrate Community

**Figure 1:**
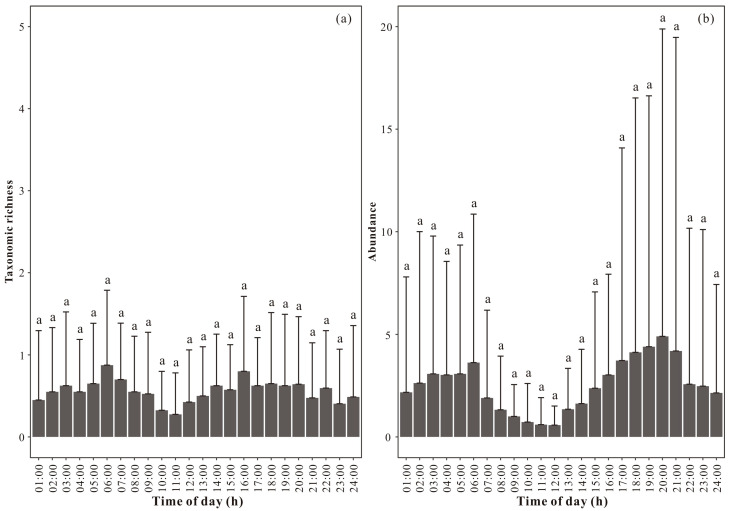
Hourly dynamics in (**a**) taxonomic richness and (**b**) abundance of the total ground-dwelling invertebrate community (mean ± STD). The different letters in the figure indicate a significant difference.

A total of 9 taxa and 1147 individuals were photographed. The largest peak in the taxonomic richness of the total ground-dwelling invertebrate community occurred at 6:00 (i.e., records from 5:00 to 6:00), and the smaller peak occurred at 16:00 (i.e., records from 15:00 to 16:00). Between 6:00 and 16:00, there was a sharp decrease in taxonomic richness, but its values did not drop to zero. The lowest values of taxonomic richness were recorded at 10:00 (i.e., records from 9:00 to 10:00) and 11:00 (i.e., records from 10:00 to 11:00) (Figure 1a). There were no significant differences during 24 h of a day for the taxonomic richness of the total ground-dwelling invertebrate community nor between any two time points of a day (Figure 1a).

The largest peak in the abundance of the total ground-dwelling invertebrate community appeared at 20:00 (i.e., records from 19:00 to 20:00), while a relatively large peak appeared at 6:00 (i.e., records from 5:00 to 6:00). Between 6:00 and 20:00, a sudden decrease in abundance was observed, but its values never reached zero. The lowest abundance values were at 11:00 (i.e., records from 10:00 to 11:00) and 12:00 (i.e., records from 11:00 to 12:00) (Figure 1b). There were no significant differences in the abundance of the total ground-dwelling invertebrate community during a 24 h period nor between any two time points of a day (Figure 1b).

**Figure 2:**
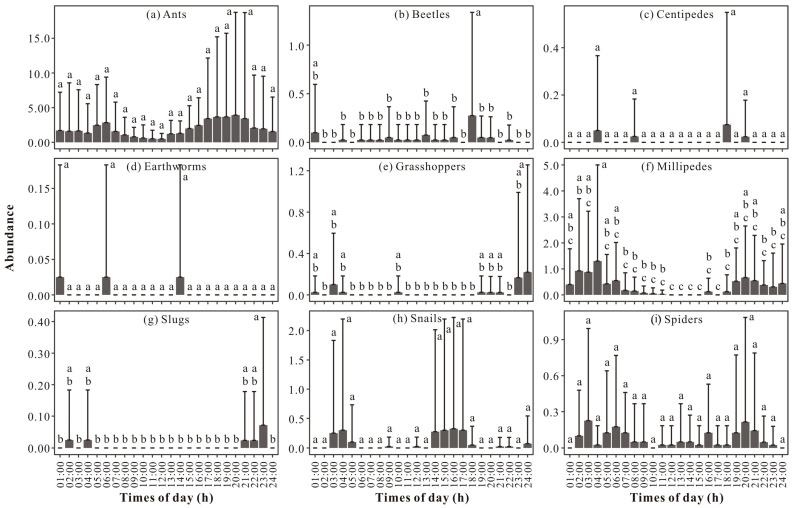
Hourly dynamics in the abundance of (**a**) ants, (**b**) beetles, (**c**) centipedes, (**d**) earthworms, (**e**) grasshoppers, (**f**) millipedes, (**g**) slugs, (**h**) snails, and (**i**) spiders (mean ± STD). The different letters in the figure indicate a significant difference.

The largest peak in millipede abundance appeared at 4:00 (i.e., records from 3:00 to 4:00), with another relatively large peak appearing at 20:00 (i.e., records from 19:00 to 20:00). Between 4:00 and 20:00, there was a relatively sharp decrease in millipede abundance. The lowest abundance values appeared at 12:00 (i.e., records from 11:00 to 12:00), 13:00 (i.e., records from 12:00 to 13:00), 14:00 (i.e., records from 13:00 to 14:00), 15:00 (i.e., records from 14:00 to 15:00), and 17:00 (i.e., records from 16:00 to 17:00), with no millipedes active at those time points. Significant differences in the abundance of millipedes (F = 2.465, *p* < 0.001) was observed during 24 h of a day. The abundance of millipedes at 4:00 was significantly higher than that at 14 other monitoring time points, except at 1:00, 2:00, 3:00, 5:00, 6:00, 19:00, 20:00, 21:00, and 24:00. Additionally, abundance at 2:00 (i.e., records from 1:00 to 2:00) was significantly higher than that at five other monitoring time points, 12:00, 13:00, 14:00, 15:00, and 17:00 (Figure 2f).

No significant differences were detected during 24 h of a day for other taxa, but significant differences were found between different time points. The abundance of slugs at 23:00 (i.e., records from 22:00 to 23:00) was significantly higher than that at 19 other monitoring time points, except at 2:00, 4:00, 21:00, and 22:00 (Figure 2g). In beetles, abundance at 18:00 (i.e., records from 17:00 to 18:00) was significantly higher than that at 22 other monitoring time points, except at 1:00 (Figure 2b). The abundance of grasshoppers at 24:00 (i.e., records from 23:00 to 24:00) was significantly higher than that at 15 other monitoring time points, except at 1:00, 3:00, 4:00, 10:00, 19:00, 20:00, 21:00, and 23:00 (Figure 2e). In the abundances of ants (Figure 2a), spiders (Figure 2i), centipedes (Figure 2c), snails (Figure 2h), and earthworms (Figure 2d), no significant differences were observed between any two time points of a day.

**Figure 3:**
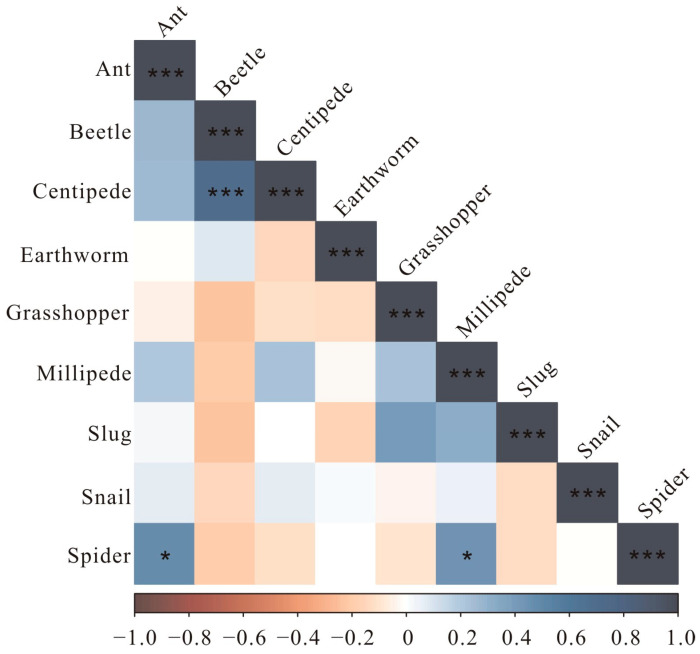
Correlation analysis between different taxonomic abundances. Pearson correlation values range from −1 to 1. The positive values are in blue, and the negative values are in red. * *p* < 0.05, *** *p* < 0.001.

Spiders and ants (r = 0.47, *p* < 0.05), spiders and millipedes (r = 0.44, *p* < 0.05), and centipedes and beetles (r = 0.70, *p* < 0.001) all exhibited positive correlations in abundance throughout a day (Figure 3).

### 3.2. Relationships between Monitoring Hour, Monitoring Day, and the Ground-Dwelling Invertebrate Community

The taxonomic richness (F = 6.144, *p* < 0.05) and abundance (F = 10.710, *p* < 0.01) of the total ground-dwelling invertebrate community were significantly negatively correlated with the monitoring Day but not with the monitoring Hour (Table 1).

The abundance of millipedes was significantly negatively and positively correlated with the monitoring Hour (F = 4.565, *p* < 0.05) and Day (F = 8.383, *p* < 0.01), respectively. Conversely, grasshopper abundance was positively and negatively correlated with the monitoring Hour (F = 4.198, *p* < 0.05) and Day (F = 8.259, *p* < 0.01), respectively. Ant abundance (F = 18.554, *p* < 0001) was significantly negatively correlated with the monitoring Day, whereas spider abundance (F = 15.589, *p* < 0.001) was significantly positively correlated with the monitoring Day (Table 1).

## 4. Discussion

### 4.1. Hourly Dynamics of the Taxonomic Richness and Abundance of the Total Ground-Dwelling Invertebrate Community

The ground-dwelling invertebrate community was active throughout the entire 24 h period of each day from August to September in the farmland. Peak taxonomic richness activity occurred from 5:00 to 6:00 and 15:00 to 16:00. Generally, the time points from 4:00 to 7:00 and 13:00 to 20:00 were highly active periods for the taxonomic richness of the total ground-dwelling invertebrate community. Conversely, the peak activity of abundance was observed between 19:00 and 20:00 and between 18:00 and 19:00. The periods from 2:00 to 6:00 and from 16:00 to 21:00 showed high activity for the abundance of the total ground-dwelling invertebrate community. Other studies have also accurately detected the active hours of the day for different invertebrate taxa in terrestrial and hydrological ecosystems through hourly investigations spanning certain periods of the day [35,36,37]. In an hourly investigation between 9:00 and 18:30 across five days in July, the abundance, species richness, and species diversity of butterfly communities peaked at periods around midday, that is, between 10:00 and 11:00, 11:00 and 12:00, and 11:00 and 12:00, respectively [38]. In another study, rove beetle communities were observed hourly from 18:00 to 6:00, with a significantly high abundance recorded between 18:00 and 21:00, after sunset [20]. In the present study, an inconsistent period was observed between the time points of high taxonomic richness and abundance of the total ground-dwelling invertebrate community, indicating that different taxa tend to be active at different hours.

Here, the lowest values for both taxonomic richness and abundance of the total ground-dwelling invertebrate community occurred at approximately 12:00. Temporal changes in the activities of certain ground-dwelling invertebrates are sensitive to temperature fluctuations [39]. For example, Enchytraeidae and earthworms are not active on the soil surface and instead move to deeper soil to avoid high-temperature stress [40,41], whereas ant species (*Aphaenogaster senilis*) avoid activity during midday to escape from high temperatures [42]. Therefore, we suggest that both the taxonomic richness and abundance of the total ground-dwelling invertebrate community responded to high temperatures at midday by pausing or decreasing their activity on the soil surface.

The taxonomic richness and abundance of the total ground-dwelling invertebrate community did not exhibit significant hourly variations across the 24 h daily period in the present study. Although previous studies have found significant diel activities in ground-dwelling invertebrates [16,17], these results could not directly confirm significant hourly variations over 24 h of a day. Additionally, no significant differences were observed between any two monitoring time points of the day, suggesting a lack of clear preferential monitoring hours for ground-dwelling invertebrates at the community level. In fact, the taxonomic richness and abundance of the total ground-dwelling invertebrate community did not significantly vary according to the monitoring Hour, unlike the monitoring Day. These results suggest that daily variations might be more obvious than hourly variations in the taxonomic richness and abundance of the total ground-dwelling invertebrate community in subtropical areas in August and September. Related studies have shown that ground-dwelling invertebrate communities exhibit significant daily [43], monthly [44], seasonal [45], and yearly variations [46]. The significant dynamics of ground-dwelling invertebrates at relatively coarse temporal scales indicate that resource and habitat partitioning, as well as biotic interactions, are important drivers of species coexistence in invertebrate communities, especially at relatively fine spatial scales [15,47,48]. However, the results of the present study suggest that there might not be a temporal partitioning of resources and microhabitats for total coexisting ground-dwelling invertebrates at the community level in farmlands. Unfortunately, we were unable to interpret the underlying processes of the nonsignificant hourly variations in the richness and abundance of the total ground-dwelling invertebrate community in the present study owing to a lack of environmental factors and species-level identification. Considering the relatively weak evidence regarding the hourly activity of the total ground-dwelling invertebrate community, we recommend conducting further experiments in various types of farmlands and during different seasons in a similar area. Additionally, more research is needed to understand the maintenance mechanisms of ground-dwelling invertebrate communities.

### 4.2. Hourly Dynamics of Abundance in Each Taxon

The hours with the highest activity of millipede abundance were from 19:00 to 6:00 in the present study, suggesting that these prefer to be active during the night time. Surprisingly, the abundance of millipedes displayed a significant hourly variation over 24 h of a day, indicating a clear hourly partitioning. Additionally, the abundance of millipedes showed significantly preferred active hours during the day, from 1:00 to 2:00 and from 3:00 to 4:00. Millipede abundance at these two periods was significantly higher than that at other time points. Although the abundance of millipedes at 3:00 was not significantly higher than that at other time points, it was still the third highest. Therefore, the preferred active period for millipede abundance in the present study spanned 3 h, ranging from 1:00 to 4:00. In contrast to our results, the majority of millipedes (*Zephronia* cf. *viridescens*) were reported to feed across all hours of the day under leaf litter in a deciduous forest [49] and a mesocosm experiment [50]. In the forest, millipedes displayed a sex-dependent walking activity, with males waking more than females throughout the day [49]. Related studies have also shown that millipedes exhibit significant seasonal dynamics [51] and diurnal rhythms (*Proteroiulus fuscus*) [52]. Additionally, the abundance of millipedes was significantly positively correlated with the monitoring Day in the present study, indicating a large variation in daily temporal resolutions. Overall, these results suggest that millipede abundance may have significant temporal dynamics at various temporal scales, and further studies are needed.

Millipede activity decreased between 6:00 and 20:00 in the present study. Considering that the abundances values of millipedes at 12:00, 13:00, 14:00, and 15:00 were zero and significantly lower than those at 4:00 and 2:00, we concluded that millipedes stopped being active on the soil surface from 12:00 to 15:00 during the experimental period in the present study. Millipedes, especially *Oxidus gracilis* in the studied farmland [40], are sensitive to temperature [53]. We suggest that millipedes were inactive or rested during periods after midday in the present study to avoid high soil temperatures or save energy. Previous studies have shown that female millipedes tend to accumulate energy across all hours of the day for reproduction; consequently, females show lower activity at night and feed more in the daytime [49,50]. In contrast, millipede males tend to spend more time crawling throughout the day [49,50]. Millipedes are the most abundant ground-dwelling invertebrates in the studied farmlands [40], playing roles both as decomposers (litter mineralization and soil enrichment) [54,55] and pests [56]. Therefore, the hourly dynamics of female and male millipedes and their influencing factors, such as biotic interactions [57], microhabitats [58], and temperatures [53], should be thoroughly studied.

Slugs were only active during the 20:00–23:00, 1:00–2:00, and 3:00–4:00 periods in the present study. The abundance of slugs from 22:00 to 23:00 was significantly higher than that at other times of the day, indicating that slugs preferred to be active during this period in the study area. The results of this study are consistent with those of previous studies on facility farmlands, in which the peak activity of slugs occurred between 22:00 and 23:00 [59]. In the same study area, slugs preferred to be active at night and were sensitive to temperature and humidity from July to September [60]. Additionally, the abundance of slugs decreased when air temperatures were higher than 25 °C in a facility farmland [61], while the growth, life-history traits, and egg hatchability of slugs (*Arion Lusitanicus*) were significantly correlated with temperature [62]. As slugs are important pests affecting various crops in the study area [60,61], the hourly dynamics of different slug species and their influencing factors should also be further considered.

Beetles were active throughout the day in the present study, but their abundance was significantly higher from 17:00 to 18:00, around sunset. Previous studies have detected various diel rhythms in beetles from different ecosystems [63]. For example, diurnal, nocturnal, and both diurnal and nocturnal beetles were observed in a Scottish farmland [63]. Furthermore, carabid beetles display diurnal or nocturnal activity patterns in lowland and alpine areas depending on their species characteristics, habitat type, and ground surface temperature [23]. Daytime factors, soil surface temperature, season, locality [64], fragmentation, and land-use changes [65] have also been reported to affect the diurnal activity of ground-dwelling beetles in forests and clear-cut areas. Therefore, soil parameters, season, habitat, and land use should be considered to understand the hourly dynamics of ground-dwelling beetles in farmlands.

In the present study, grasshoppers mostly appeared at night, with a significantly higher abundance from 23:00 to 24:00. The bladder grasshopper (*Bullacris unicolor*) sings at different times during the night [66]. Grasshopper males distributed in northern areas have been reported to sing before dawn, whereas those in southern areas sing throughout the night [66]. Identifying species and analyzing the traits of grasshoppers will aid in understanding their diel dynamics within a daily 24 h period.

The abundances of ants, spiders, centipedes, snails, and earthworms did not vary significantly during 24 h of a day in the present study, and none of them exhibited significantly preferred activity hours. However, previous studies have reported significant diel activity patterns. The diel dynamics of ants have been extensively studied; for example, certain ant species/populations forage all day [1], whereas others only forage either nocturnally [2] or diurnally. Within an orchard spider community, different families showed significantly different preferred active periods during the day, with the abundances of Anyphaenidae, Philodromidae, and Thomisidae peaking at 01:00 ± 3 h (mean ± SE), 23:00 ± 5 h, and 24:00 ± 6 h, respectively [14]. Snails are active and damage crops at night in farmlands in the study area, but they are also active during the day when the weather is cloudy [67]. A study on earthworms showed that certain individuals (*Lumbricus terrestris* L.) dispersed on the soil surface at night in a manipulated experiment using infrared-sensitive web cameras [68], while another study found that two epigeic earthworm species (*Lumbricus rubellus* and *Dendrobaena octaedra*) benefited from the absence of diurnal fluctuations in forest soil [69]. However, the abundance of ants, spiders, millipedes, and grasshoppers was significantly correlated with the monitoring Day in the present study, indicating that daily variation, rather than hourly variation, might exist for these taxa. Overall, the different findings of the previous and present studies evidence the complicated hourly dynamics of these taxa in the field.

A Pearson correlation analysis revealed significant coexisting times in the behaviors of spiders with ants, spiders with millipedes, and centipedes with beetles over a 24 h period. Spiders and ants, often considered potential competitors, have been known to prey on each other [70,71]. In tropical rain forest canopies, a negative correlation was observed between ants and spiders, with their exclusive spatial distributions attributed to antagonistic interspecific interactions [72]. Furthermore, the abundance of web-building spiders was negatively impacted by ants, while ants of the *Formica* genus were negatively affected by wandering spiders in grasslands, suggesting that intraguild interactions are key drivers of their relationship [71]. Although a negative impact of ants on spider abundance was noted in Douglas fir canopies due to interference competition resulting from ant foraging and aphid-tending activities, no exploitative competition for prey was found between them [70]. Spiders, centipedes, and some beetles are generalist predators [73]. Previous research has documented that spiders prey on millipedes in the field, yet their temporal relationship at finer scales, such as hourly intervals, remains largely unexplored [74]. A negative co-occurrence pattern was noted between beetles (*Pterostichus stygicus*) and centipedes (*Scolopocryptops sexspinosus*) under artificial cover objects both in field and laboratory settings, suggesting a potential competition between these species for shared microhabitat and prey [73]. The positive correlations detected in our study between the abundances of spiders and ants, spiders and millipedes, and centipedes and beetles during a 24 h period do not contradict previous findings. Instead, they underscore the close interspecific relationships and potential competition for food resources, microhabitats, and activity spaces, as well as the roles of these taxa as predators, within the ground-dwelling invertebrate community in farmland. Thus, studying the fine temporal dynamics (e.g., hourly in the present study) of different ground-dwelling taxa offers a novel approach to understanding interspecific relationships and their contributions to species coexistence and community maintenance.

Additionally, the results of this study suggest that significant hourly variations during 24 h of a day were not general at both the taxon (except for millipedes) and community levels for ground-dwelling invertebrates in farmland, whereas significantly preferred active hours existed at the taxon rather than community levels. Similarly, significant wave-like fluctuations have been observed in certain bacterial-feeding nematode populations but not for nematode communities in microcosm experiments at a fine temporal resolution [75]. These results indicate that the abundance and activity of ground-dwelling invertebrates exhibit different temporal strategies within a daily 24 h period at the taxa and community levels. Therefore, studies on hourly or diel dynamics at the species/population level in ground-dwelling invertebrates are not sufficiently robust to explain these dynamics at the community level. Studies on hourly or diel dynamics at both the species/population and community levels are necessary to understand the underlying processes of soil biodiversity, protect agricultural soil biodiversity, and understand the responses of soil biodiversity to climate change and human management.

The lack of finer taxonomic classification at the species level limits our ability to fully explain the hourly dynamics in the richness and abundance of the total ground-dwelling invertebrate community and the abundance of each taxon. Currently, there is no consensus on the optimal taxonomic resolution for biodiversity monitoring and assessment [76]. While some studies suggest the greatest benefits are at the genus or species levels [77,78], other research has shown that a coarse taxonomic classification, such as the family and suborder for Oribatid mites or the order and class for Isopoda, Diplopoda, etc., is sufficient or may even be superior [79,80]. Given the notably scarce biodiversity information at finer temporal scales for most ground-dwelling invertebrates, focusing on a relatively coarse taxonomic classification is one approach to overcome this limitation [81]. A previous study at Tiansheng Farm showed that coarse taxonomic classification, particularly at the family level, is effective for farmland biodiversity assessment [82]. Therefore, the coarse classification used in our study still provides valuable insights for assessing the hourly dynamics of ground-dwelling invertebrates in field biodiversity monitoring. Furthermore, with the rapid advancement of AI technologies and their application in invertebrate identification [83], such as the SqueezeNet transfer learning model for the tiger beetle [84] and faster region-based convolutional networks for Collembola [85], and their application in invertebrate identification offer solutions to these challenges when integrated with morphological classification methods. Additionally, considering the trophic relationships among collected taxa and defining their roles in soil processes are essential to understand the hourly dynamics of ground-dwelling invertebrate communities in farmland. We believe that combining infrared camera traps with AI technologies holds significant potential to address these challenges.

Additionally, the taxonomic richness, abundance of the total ground-dwelling invertebrate community, and abundances of ants, spiders, millipedes, and grasshoppers were significantly correlated with the monitoring Day, indicating that factors occurring at a day temporal resolution may affect hourly variations. According to previous studies, various factors affect the diel variations of ground-dwelling invertebrate communities, including light intensity [86], temperature [39], precipitation [87], weather [20], food resources [6,88], farmland management [14], and vegetation [89]. Thus, we suggest that these important factors, which occur at hourly and daily temporal resolutions, should be studied further to understand the fine temporal dynamics of ground-dwelling invertebrate communities in farmlands.

## 5. Summary

In a study conducted in a farmland field in Ningbo City, southeastern China, the hourly dynamics of ground-dwelling invertebrate communities were analyzed over 42 days (1008 h), utilizing 222,912 images captured using infrared camera traps (ICTs). We observed that the taxonomic richness and abundance of the entire community did not exhibit significant hourly variations within a 24 h period or between any two time points during a day. However, higher levels of taxonomic richness and abundance were noted in the morning, afternoon, and before midnight, though these were not statistically significant. Among the different taxa, only millipedes displayed significant variations in abundance, being most active during the predawn period. Conversely, slugs, beetles, and grasshoppers were significantly more active around sunset and near midnight, while ants, spiders, centipedes, snails, and earthworms did not show significant preferences for active hours. Generalist predators maintained similar activity patterns throughout the day. The results indicate that significant variations over a 24 h period are not typical for the total ground-dwelling invertebrate community and most taxa in subtropical farmlands. Most taxa exhibited preferred active hours of the day, unlike the total ground-dwelling invertebrate community. This study, however, faced limitations due to the coarse classification of taxa and the absence of environmental factors, which restricted a deeper understanding of the hourly dynamics. Future research should incorporate finer taxonomic classification and consider factors such as hourly temperature and humidity, soil parameters, and biotic interactions. This study offers a valuable methodology for monitoring hourly changes in invertebrate communities in farmlands, providing detailed temporal data essential for biodiversity conservation. It also contributes to understanding the responses of these communities to climate change and human management practices.

## Figures and Tables

**Table 1:** Effects of monitoring Hour and Day on taxonomic richness and abundance of the total ground-dwelling invertebrate community and abundance of each taxon.

Taxa	Monitoring Hour			Monitoring Day		
	F	*P*	*r*	F	*p*	*r*
Taxonomic richness	0.001	0.973	<0.001	6.144	0.013	−0.001
Total abundance	2.101	0.147	0.008	10.710	0.001	−0.010
Ants	3.643	0.056	0.01	18.554	<0.001	−0.013
Beetles	0.153	0.696	<0.001	0.008	0.929	−0.00001
Centipedes	0.003	0.954	<0.001	1.538	0.215	−0.00007
Earthworms	1.928	0.165	−0.00006	0.199	0.655	<0.001
Grasshoppers	4.198	0.041	0.0005	8.259	0.004	−0.0004
Millipedes	4.565	0.033	−0.002	8.383	0.004	0.002
Slugs	2.089	0.148	<0.001	0.993	0.319	<0.001
Snails	0.017	0.895	−0.0001	1.205	0.272	0.0005
Spiders	0.484	0.487	−0.0002	15.589	<0.001	<0.001

## Data Availability

The raw data supporting the conclusions of this article are included in the article materials; further inquiries can be directed to the corresponding author.
